# Immediate hypersensitivity reactions to iodinated contrast media: The diagnosis of allergy by skin testing

**DOI:** 10.1002/clt2.12214

**Published:** 2022-12-04

**Authors:** Juliette Caron, Sahara Graf, Christine Delebarre‐Sauvage

**Affiliations:** ^1^ Service d'allergologie et d’éducation thérapeutique Hôpital Saint Vincent‐de‐Paul Lille Cedex France; ^2^ Délégation à la Recherche Clinique et à l’Innovation Unité de Biostatistiques Hôpital Saint Philibert Lomme Cedex France

**Keywords:** allergy, hypersensitivity, iodinated contrast media, skin tests

## Abstract

Among 74 patients with an immediate hypersensitivity reaction (IHR) to iodinated contrast media (ICM), the rate of allergic patients confirmed by positive prick test or diluted intradermal test (IDT) was 8.1%. 12.5% of re‐exposed patients had a recurrent IHR despite negative skin tests. Investigations on pure IDT to ICM and development of drug provocation test may provide additional safety nets to uncover recurrent ICM reactors. Agreements among allergists are needed to unify practices.

**Abbreviations**
DPT: drug provocation test
ICM: iodinated contrast media
IDT: intradermal test
IgE: immunoglobulin E
IHR: immediate hypersensitivity reaction
NPV: negative predictive value
PT: prick test
ST: skin test

To the Editor,

In the past, most immediate hypersensitivity reactions (IHR) due to iodinated contrast media (ICM) were considered to be non‐immunological. Recently studies have demonstrated an authentic immunological mechanism mediated by immunoglobulin E.^1^ For these allergic patients, the re‐exposure to ICM could raise a life‐threatening risk. In case of IHR to ICM, some allergy units considered positive skin tests (ST) to be reliable enough to assert an allergy: undiluted prick test (PT) or diluted intradermal test (IDT).^2^, ^3^ However, some studies have shown that negative ST patients were not safe from a recurrent IHR to ICM.^4^

We carried out a retrospective study to determine the outcome of re‐exposure to ICM for the patients tested in our allergy unit. All consecutive patients from January 2010 to June 2019 with a prior IHR (<1 h) to ICM were included. We performed undiluted PT then diluted IDT, ranging from 1:100,000 to 1:10, not depending on the severity of reactions. We used the following ICM: iobitridol 300 mg/ml, iohexol 350 mg/ml, iopamidol 300 mg/ml, iomeprol 300 mg/ml, ioversol 350 mg/ml, and iodixanol 320 mg/ml. We evaluated ST positivity according to the standards in force at 20 min reading.^2^ If the culprit ICM was known, a panel of ICM including the culprit one was tested. We allowed the use of ICM with negative ST after the allergy testing, save the culprit ICM if the initial reaction was ≥ grade II. If the culprit ICM was unknown, a panel of ICM was tested. We allowed the use of all ICM with negative ST. In all cases, we forbid the use of all ICM with positive ST. In August 2019, all patients were called back to ascertain information concerning any new injection of ICM. If a new IHR occurred at the re‐exposure, the clinical severity was recorded.

The study population was composed of 74 patients (Table 1). A median of five ICM and six ICM were tested among patients whose culprit ICM was known and unknown, respectively. 8.1% [95% CI 3.8; 16.6] of patients had at least one positive ST. The rate of allergic patients increased with the clinical severity.

**TABLE 1:** Description of the population

	Missing values *n*	Cohort *n* (%)	Positive‐skin‐test patients *n* (%)	Negative‐skin‐test patients *n* (%)	*p*
Number (%)	0	74	6 (8.1)	68 (91.9)	
Female, *n* (%)	0	47 (63.5)	4 (66.7)	43 (63.2)	1
Age (years), mean ± standard deviation, *n* (%)	0	58 ± 12	55 ± 7	58 ± 13	0.24
History of asthma, *n* (%)	4	9 (12.9)	1 (16.7)	8 (12.5)	0.58
History of chronic obstructive pulmonary disease, *n* (%)	4	4 (5.7)	0 (0)	4 (6.2)	1
History of atopic dermatitis, *n* (%)	4	6 (8.6)	0 (0)	6 (9.4)	1
Suspected drug allergy, *n* (%)	2	34 (47.2)	2 (33.3)	32 (48.5)	0.68
History of cardiovascular disease (smoking excluded), *n* (%)	2	48 (66.7)	3 (50)	45 (68.2)	0.39
Active smoker or ceased less than 3 years previously, *n* (%)	28	15 (32.6)	1 (33.3)	14 (32.6)	1
History of cancer, *n* (%)	4	23 (32.9)	3 (50)	20 (31.2)	0.39
History of chronic kidney failure, *n* (%)	4	2 (2.9)	1 (16.7)	1 (1.6)	0.17
Family history of atopic dermatitis, *n* (%)	44	2 (6.7)	0 (0)	2 (7.1)	1
Family history of allergic rhinitis, *n* (%)	4	6 (8.6)	1 (16.7)	5 (7.8)	0.43
Conversion enzyme inhibitor treatment, *n* (%)	5	14 (20.3)	0 (0)	14 (22.2)	0.33
Angiotensin II receptor blocker treatment, *n* (%)	5	14 (20.3)	1 (16.7)	13 (20.6)	1
Beta blocker treatment, *n* (%)	5	20 (29)	1 (16.7)	19 (30.2)	0.66
ICM involved	0				0.81
Iobitridol, *n* (%)		4 (5.4)	0 (0)	4 (5.9)	
Iodixanol, *n* (%)		13 (17.6)	0 (0)	13 (19.1)	
Iohexol, *n* (%)		4 (5.4)	0 (0)	4 (5.9)	
Iomeprol, *n* (%)		1 (1.4)	0 (0)	1 (1.5)	
Iopamidol, *n* (%)		1 (1.4)	0 (0)	1 (1.5)	
Ioversol, *n* (%)		8 (10.8)	1 (16.7)	7 (10.3)	
Unknown, *n* (%)		43 (58.1)	5 (83.3)	38 (55.9)	
Type of radiological examination	11				0.42
Computed tomography arthrography, *n* (%)		3 (4.8)	0 (0)	3 (5.2)	
Cholecystography, *n* (%)		1 (1.5)	1 (20)	0 (0)	
Computed tomography colonography, *n* (%)		2 (3.2)	0 (0)	2 (3.4)	
Coronary angiogram, *n* (%)		3 (4.8)	0 (0)	3 (5.2)	
Coronary computed tomography, *n* (%)		1 (1.6)	0 (0)	1 (1.7)	
Hysterography, *n* (%)		2 (3.2)	0 (0)	2 (3.4)	
Intra‐articular injection: Spine/knee, *n* (%)		9 (14.3)	0 (0)	9 (15.5)	
Computed tomography (other than spine)		37 (58.7)	4 (80)	33 (56.9)	
Computed tomography (spine), *n* (%)		1 (1.6)	0 (0)	1 (1.7)	
Urography, *n* (%)		4 (6.3)	0 (0)	4 (6.9)	
Route of administration	11				0.24
Intravascular, *n* (%)		47 (74.6)	4 (80)	43 (74.1)	
Intra‐articular, *n* (%)		12 (19)	0 (0)	12 (20.7)	
Intracavitary, *n* (%)		3 (4.8)	1 (20)	2 (3.4)	
Intravenous + oral, *n* (%)		1 (1.6)	0 (0)	1 (1.7)	
Ring and Messmer clinical grade	0				0.096
I, *n* (%)		39 (52.7)	1 (16.7)	38 (55.9)	
II, *n* (%)		26 (35.1)	3 (50)	23 (33.8)	
III, *n* (%)		9 (12.2)	2 (33.3)	7 (10.3)	
Time interval from reaction to skin testing (months), mean ± SD	9	111 ± 124	225 ± 166	91 ± 105	0.065

Forty patients were re‐exposed to ICM. Thirty five patients were re‐injected once. Five patients were re‐injected twice or more. 54.1%, 60% and 62.2% of patients whose initial reaction was, respectively, grade I, grade II and grade III were re‐injected.

Five re‐exposed patients (12.5%) had a recurrent IHR after a new ICM administration despite negative ST for the ICM agent (Table 2). The NPV of the allergy work‐up for predicting an IHR to ICM was 88.1% [95% CI 74.4; 96]. Five IHR among 42 re‐exposures were observed. Four out of these five IHR were less or equally severe compared with the initial reaction. For one patient re‐exposed accidently to iodixanol (initial reaction to iodixanol), the IHR was more severe than previously: grade IV versus grade III. During the allergy work‐up, this patient had negative ST to iodixanol, latex, povidone‐iodine. As the initial reaction was severe, we contraindicated the use of iodixanol. During an urgent coronary angiogram, the patient accidently received iodixanol and experienced a grade IV reaction 1 h after. The coronary angiogram was strictly normal. Serum tryptase was unmeasured.

**TABLE 2:** Re‐exposure to ICM: 5 patients out of 40 with a recurrent IHR to ICM after the allergy work‐up

Sex	Age at skin tests (years)	ICM (administration route)	Grade of initial reaction	Time from initial reaction to skin tests (months)	Skin tests	Year of skin tests	ICM reintroduced (route of administration)	Grade
Male	66	Unknown (IA)	II	60	Negative	2016	Iohexol (IA)	II
Female	51	Ioversol (IV)	III	6	Positive IDT Iodixanol Iohexol Ioversol	2017	Iopamidol (IV)	II
Male	72	Ioversol (IV)	II	5	Negative	2017	Iohexol (IV)	II
Female	69	Iodixanol (IV)	III	11	Negative	2018	Iodixanol (IV)	IV
Male	58	Iodixanol (IV)	I	72	Negative	2018	Iohexol (IV)	I

Abbreviations: IA, intra‐articular; IDR, intradermal reaction; IV, intravenous.

In our study, IHR at ICM re‐exposure were frequent (12.5%). The NPV of the allergy work‐up for predicting an IHR to ICM was 88.1% [95% CI 74.4; 96]. This rate is near than reported by Schrijvers who found 94.2% [89.6; 97.2].^5^ Despite no proof of an immunological mechanism during skin testing, we found that these reactions could be as severe as initial ones. How to explain these recurrences? Diluted IDT might lack sensitivity to identify IgE‐mediated reactions to ICM or these reactions hide a non‐IgE‐mediated underlying mechanism.

In any case, the priority remains to uncover recurrent ICM reactors. The use of pure IDT may have a role to play,^1^, ^6^ on condition that the false positive rate remains low. As pure IDT to ICM are relatively irritant, more studies are needed on this subject.

Other than skin testing, few other tools exist to study IgE‐mediated mechanisms to ICM. The Basophil Activation Test is sometimes used in studies to prove IgE‐mediated mechanism. However, its diagnostic values remain unknown.^2^ As pathophysiology concerning IHR to ICM remains unclear, biological tests might lack sensitivity to identify an allergy.

Some authors propose a drug provocation test (DPT) as a complementary test in addition to ST.^2^, ^7^ This clinical test is usually the gold standard for identifying drug‐induced reactions.^8^ So far, the use of DPT for ICM has been under debate.^2^ Some authors do not include DPT in there management,^3^ while others seem to propose it systematically.^4^, ^8^ DPT are sometimes proposed only in case of a positive ST to ICM in order to confirm the absence of cross‐reactivity to an alternative ICM.^2^, ^7^ DPT protocols are not standardized.^2^

As our study was retrospective, we acknowledge the presence of potential bias. We concede the only tool we had to confirm IgE‐mediated allergic mechanism was skin testing. One can reproach a lack of sensitivity concerning our allergy work‐up, because the time interval between the initial reaction and skin testing was long (111 months). Some authors considered this long interval could lead to negative ST results and drug tolerance in case of re‐exposure.^2^, ^8^ Brockow found that 50% of patients who underwent ST for ICM within 6 months had positive tests versus only 22% if tested later than 6 months.^9^ Based on this study, skin testing is recommended between two and 6 months after a reaction to ICM.^2^, ^8^ Paradoxically, in our study, the average time interval between the initial reaction and skin testing was longer for patients with positive ST than those with negative ST, 225 versus 91 months respectively. Our result seems to be contradictory to Brockow's findings.

Investigations on pure IDT to ICM and development of DPT may provide additional safety nets to uncover recurrent ICM reactors. Agreements among allergists are needed to unify practices.

## AUTHOR CONTRIBUTIONS

**Juliette Caron**: Conceptualization (Lead); Data curation (Equal); Investigation (Lead); Methodology (Lead); Project administration; Lead, Resources (Equal); Supervision (Equal); Validation (Equal); Visualization (Lead); Writing – original draft (Lead); Writing – review & editing (Lead). **Sahara Graf**: Formal analysis (Lead), Funding acquisition (Equal); Software (Lead); Validation (Equal). **Christine Delebarre‐Sauvage**: Funding acquisition (Lead); Investigation (Equal); Project administration (Supporting); Resources (Equal); Supervision (Equal); Validation (Equal).

## CONFLICT OF INTEREST

The authors declare that they have no competing interests.

## FUNDING INFORMATION

Catholic Institute of Lille Hospital Group.
